# Biodegradable Flame Retardants for Biodegradable Polymer

**DOI:** 10.3390/biom10071038

**Published:** 2020-07-11

**Authors:** Muhammad Maqsood, Gunnar Seide

**Affiliations:** Aachen Maastricht Institute for Biobased Materials, Faculty of Science and Engineering, Maastricht University, Urmonderbaan 22, 6167 RD Geleen, The Netherlands; muhammad.maqsood@maastrichtuniversity.nl

**Keywords:** polylactic acid, biodegradability, sustainability, renewable resources

## Abstract

To improve sustainability of polymers and to reduce carbon footprint, polymers from renewable resources are given significant attention due to the developing concern over environmental protection. The renewable materials are progressively used in many technical applications instead of short-term-use products. However, among other applications, the flame retardancy of such polymers needs to be improved for technical applications due to potential fire risk and their involvement in our daily life. To overcome this potential risk, various flame retardants (FRs) compounds based on conventional and non-conventional approaches such as inorganic FRs, nitrogen-based FRs, halogenated FRs and nanofillers were synthesized. However, most of the conventional FRs are non-biodegradable and if disposed in the landfill, microorganisms in the soil or water cannot degrade them. Hence, they remain in the environment for long time and may find their way not only in the food chain but can also easily attach to any airborne particle and can travel distances and may end up in freshwater, food products, ecosystems, or even can be inhaled if they are present in the air. Furthermore, it is not a good choice to use non-biodegradable FRs in biodegradable polymers such as polylactic acid (PLA). Therefore, the goal of this review paper is to promote the use of biodegradable and bio-based compounds for flame retardants used in polymeric materials.

## 1. Introduction

Fire hazards have become quite severe due to the invasion of polymers in our daily lives [1]. Undeniably, the majority of the frequently used polymers not only burn quite briskly and release large amounts of smoke and heat but also melt vigorously, which promotes further fire propagation [2]. Numerous financial losses and personal deaths have been reported every year due to fire propagation and the smoke thus produced, which contains toxic substances, causing severe health problems for society [3]. According to the International Association of Fire and Rescue Services (CTIF), approximately 2.0–2.5 million fire accidents were reported in Europe in 2018 resulting in 20,000–25,000 fire deaths, while 60% of the deaths were caused by smoke inhalation [4]. Therefore, in developed countries, fire regulations are becoming more stringent and generally operate using two different approaches; by implementing the safety measures such as the use of smoke detectors and fire protective equipment and by using those materials that contribute to fire as little as possible [5].

Generally, flame-retardant additives are used in polymers to improve their flame retardancy against fire hazards [6]. The fire hazards from a particular polymer can be judged by certain predefined parameters, such as time to ignition, heat release rate, fire propagation rate, amount of smoke and CO_2_ release, and the toxicity of the byproducts. Flame retardants are of different types and are generally classified based on their mode of action, chemical nature, and working and protection mechanism [7]. Halogen-containing flame retardants generally operate in the gaseous phase by terminating the free radicals involved in the combustion process. They are found to be very effective at very low additive concentrations in the polymer, but at the same time, they release toxic substances during emission and, hence, have been abandoned in most developed countries. Halogen-containing flame retardants have been gradually replaced with halogen-free flame retardants in the last couple of decades [8].

As a replacement for halogen-containing flame retardants, mineral-hydrated fillers such as aluminum tri-hydroxide (ATH) and magnesium hydroxide (Mg(OH)_2_) are used as halogen-free flame retardants [9]. However, their effectiveness is mainly dependent on higher loading concentration in the polymer, as quite often the loading concentration of such additives exceeds 40–50 wt % in the polymer in order to achieve a certain level of flame retardancy. On the one hand, such a high loading concentration of these mineral-hydrated fillers can increase flame retardancy up to the required level, but on the other hand, their mechanical properties could be affected due to which sometimes it becomes difficult to further process them [10]. Nanoparticles have been found to be very effective in flame retardant formulations, but they are seldom used alone. They are always used in combination with other materials and act as an efficient synergist. If they are well dispersed in the matrix, even very low loading concentration (1–5 wt %) can be very effective [11]. They have been found to be very efficient in lowering the peak heat release rate measured by cone calorimetry. Nanoparticles are of various types, but carbon nanotubes and clays are the most studied nanoparticles in flame retardancy [12]. Phosphorous-based flame retardants mostly promote char formation but they are also known as flame inhibitors [13]. Charring on the polymer surface not only reduces the quantity of fuel required to sustain flame but also acts as physical barrier, which restricts the polymer from further burning and from external heat flux. When the char layer swells, it becomes even more effective in restricting the passage of heat through the underlying material [14]. Such systems are known as intumescent and they are based on halogen-free flame retardants. The loading concentration of phosphorous based flame retardants in intumescent system lies in the range of 15–25 wt %, and they are not only effective but at the same time non-toxic to the environment [15].

Most of the flame retardants used in polymers are non-biodegradable and therefore tend to accumulate in the environment and are persistent [16]. Due to this non-biodegradability of halogenated FRs, certain biotic or abiotic processes can occur in specific environmental conditions. Biotic processes are the processes where bioaccumulation of the toxic substances and entrance into the food chain may occur and consequently may affect plants, humans, and wildlife [17]. Abiotic is a chemical process where photo-degradation and decomposition of the material at elevated temperatures and reactions with other compounds may occur that can change the materials intrinsic properties [18]. Moreover, non-biodegradable halogenated FRs may find their way into the environment through wastewater streams of the industries that produce them or through adsorption into the dust particles at the manufacturing facilities where they are incorporated directly to various products [19]. Once these FRs enter the environment, they can easily attach to any airborne particle and can travel distances far from their emission or production sites. Hence, traces of such non-biodegradable FRs can be found in freshwater, food products, ecosystems, or can even be inhaled if they are present in the air [20]. On the other hand, biodegradable FRs are the one that can be degraded by microorganisms in the soil or water and result in mineralization. Therefore, one solution of this problem is to use flame retardants that are biodegradable. This review paper covers three main aspects. The first portion of this review covers conventional approaches to improve the flame retardancy of polymers. In the second portion, different types of flame retardants and their mechanism of actions are discussed. In the third portion, potential of bio-based and biodegradable additives in flame retardant applications are highlighted.

## 2. Polylactic Acid (PLA)

Polylactic acid (PLA) is a bio-based polymer derived from renewable resources and can be degraded in the soil by microorganisms under certain conditions of temperature and humidity [21]. The feedstock for PLA is obtained from natural and sustainable resources such as corn starch [22]. The main producers of PLA are NatureWorks LLC (Minnetonka, MN, USA), WeforYou GmbH (Graz, Austria), Evonik Industries AG (Essen, Germany) and Total-Corbion NV (Gorinchem, The Netherlands) and the global PLA market is expected to become more than $5 billion USD by 2020 [23]. The global production volume of bioplastics in 2019 was 2.11 million tonnes according to the latest market data collected by European Bioplastics e.V. (Berlin, Germany) and Nova Research Institute GmbH (Hurth, Germany), out of which biodegradable plastics such as PLA and starch blends accounts for over 1 million tonnes [24]. PLA is recognized to be the first melt-process-able synthetic fiber derived from 100% natural resources and hence can be degraded under suitable conditions [25]. In addition to these eco-friendly benefits, PLA also offers decent performance in technical applications owing to its comparable mechanical properties to that of petroleum-based polyethylene terephthalate (PET) [26]. However, PLA is less flammable than other synthetic thermoplastics such as PET, with less visible smoke on burning and a lower peak heat release rate (pHHR) [27]. The physical and fire related properties of PLA polymer are compared with other commercial polymers such as PET, polyamide 6 (PA6), and polypropylene (PP) in Table 1.

PLA is aliphatic polyester synthesized from lactic acid building blocks [29]. In 1932, low molecular weight PLA was developed by Carothers et al. [30]. Traditionally, PLA was known for biomedical applications due to its biocompatibility; however, in the recent past, the innovation of some novel polymerization techniques led to the manufacturing of high molecular weight PLA that broadened the scope of PLA applications ranging from packaging to high-tech applications [31]. Melt processing is considered to be the most economically viable option for filament production [32]. In this technique, the polymer is heated above its melting point, and filaments are formed followed by hot drawing to obtain the desired mechanical properties [33]. For the optimization and smooth running of the process, the understanding of rheological behavior, crystallization, and thermal properties of the polymer is mandatory.

**Figure 1:**
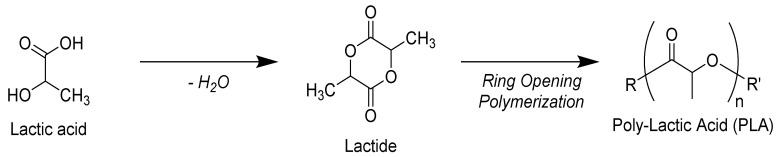
Ring opening polymerization route for the production of polylactic acid.

Lactic acid is the basic building block for polylactic acid, which is manufactured by transforming starch or sugar through bacterial fermentation or by the petrochemical route. By the poly-condensation reaction, lactic acid can be transformed directly to polyesters due to the presence of carboxyl and hydroxyl groups. However, the conventional poly-condensation reaction results in lower molecular weight of this polymer, unless organic solvents are involved in the process. Though the ring opening polymerization technique, a high molecular weight of polylactic acid can be achieved [34]. The production route of polylactic acid by ring opening polymerization is shown in Figure 1.

## 3. Flame Retardants

Carbon is the fundamental element in all types of organic polymers [35]. Carbon–carbon bonds are the backbones of each polymer, whereas some polymers also contain carbon–oxygen and carbon–nitrogen bonds depending on the presence of heteroatoms in the main chains. Upon exposure to adequate amount of heat, all type of organic polymers can be thermally decomposed. The heat supplied is absorbed as long as the carbon–carbon, carbon–oxygen, and carbon–nitrogen bonds stay in contact, but after the bonds are broken, volatile gases are emitted that act as fuel to the fire [36]. The thermal decomposition of a polymer is dependent on various factors such as the composition of a polymer, the conditions at which the polymer is degraded, and the presence of additives in the polymer. Although the degradation and stability of each polymer is dependent on the factors discussed above, but approximately all polymers tend to decompose within the temperature range of 250–450 °C [37]. Therefore, flame-retardant additives are incorporated in a polymer to improve its flame retardancy.

The task of a flame retardant is to interfere with polymer ignition and to make it less flammable by intervening in a process, by which burning phenomenon takes place [38]. Their role is to prolong the resistance to burning rather than entirely eradicating the flammability of the polymers, as all polymers are prone to ignition at extreme temperature conditions. However, by the right composition of additives, a polymer can become flame-retardant, and the ignition of a polymer can be prolonged. There is no universal flame retardant available that can be used for all types of polymers due to their different chemical compositions and different requirements for each targeted application [39]; the requirements for a foam application will be different to the requirements for molding sheets or for textiles. In short, each flame retardant is designed for a particular end use, and their selection is dependent on the type of fire hazards. Flame retardants are generally assessed by considering their performance against (a) heat release rate, (b) flame spread rate, (c) ignition of a polymer, and (d) formation of smoke or toxic gases [40]. Therefore, to get the desired results, a careful selection of a flame retardant is necessary. Sometimes more thermally stable polymer together with a flame retardant is preferred to control fire hazards; however, this approach is not so convincing because, thermally stable polymers usually contain high fluorine content (which is toxic) and are somewhat difficult to process and more costly [41].

**Figure 2:**
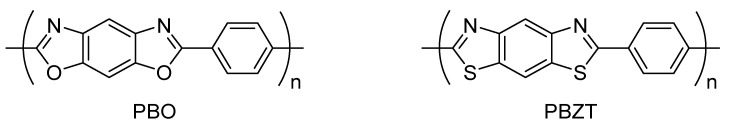
Chemical structures of inherently flame-retardant polymers (polyphenylene-benzobisoxazole (PBO) and polyphenylene-benzobisthiazole (PBZT)).

Fire propagation can be inhibited/suppressed by any of the following four approaches, i.e., (a) by using inherently flame retardant polymers (high performance polymers); (b) by chemically modifying the polymer structure; (c) by a surface treatment to the polymer; (d) by integrating fire retardants in the polymers by compounding. These approaches are briefly discussed. High-performance or inherently flame-retardant polymers such as polyphenylene-benzobisoxazole (PBO) also known as Zylon (commercial name) or polyphenylene-benzobisthiazole (PBZT) possess excellent flame-retardant properties but are not suitable for every application due to their higher cost and aging problems [42]. The chemical structures of PBO and PBZT are shown in Figure 2.

In the second approach, the monomer of a polymer is chemically modified to produce a flame-retardant polymer, such as Trevira CS [43]. It is a chemically modified PET fiber that possesses intrinsic flame-retardant properties and therefore does not require external treatment to impart flame retardancy. In the third approach, a polymer surface is coated to make it flame-retardant. This method is easy to implement and offers quite a few benefits. The biggest advantage of using this method is that flame retardancy can be imparted to a polymer without altering its intrinsic mechanical characteristics. This method can be implemented in various types of materials such as, polymers, metals, wood and textiles. There are various technologies that are used to modify the surface of a polymer and cold plasma is one of them. The surface of a material is treated to achieve desired functional properties without altering its intrinsic properties. Surface functionalization reactions are stimulated on the surface of a material by using cold plasma technology or tiny layers of organic or inorganic materials are deposited by combining the molecular fragments on materials surface by reactive species of cold plasma technology [44]. In the fourth approach, flame-retardant additives are directly incorporated to the polymers by melting and adding the required percentage of additives to form FR composites. This approach is generally favored because it provides a balance between the required flame-retardant properties and the cost occurred in the process [45]. Moreover, this approach is quite flexible in order to design polymers with desired multifunctional properties and can be used at the industrial scale as well.

Flame-retardant additives, depending on their type and composition, behave differently with the polymer as most of them interact chemically with the polymer while some interact physically as well to interfere the combustion process. There are different stages of a combustion process, starting from heating the polymer, its decomposition, ignition, and flame spreading in the polymer. Not all FR additives interfere the combustion process at same stage; some are designed to interfere before polymer decomposition, some are intended to suppress the combustion before ignition, and some are aimed to prevent the flame spreading in the polymer [46].

## 4. Action Mechanism of Flame Retardants

Since flame retardants interfere in more than one stage of the combustion process, their action mechanism is mainly dependent at which stage of combustion they interfere with [47]. The action mechanisms of different types of flame retardants are discussed below.

### 4.1. Dilution of Volatile Products

Alumina trihydrate (Al_2_O_3_·3H_2_O) is a compound used for the dilution of volatile products since it starts breaking down to water molecules and aluminum oxide at 175–210 °C [48]. Moreover, this compound is environmentally safe and not very expensive, so it decreases the overall production cost [49]. However, the drawback of this compound is its very low decomposition temperature and the fact that it requires relatively higher loading concentration (wt %) in the polymer to induce fire retardancy. To overcome these drawbacks, magnesium hydroxide Mg(OH)_2_ has recently been used for the dilution of volatile products since it operates in a similar way to alumina trihydrate but with higher decomposition temperature (280–340 °C). Hence, Mg(OH)_2_ can be used for polymers that are processed above 250 °C without the problem of early decomposition during melt processing [50]. However, a relatively higher loading concentration (40–50 wt %) of the compound is still required to impart acceptable degree of flame retardancy in the polymer.

### 4.2. Inhibition of Vapor Phase Combustion

For vapor phase combustion to take place, volatile products are required that are produced by oxidative thermal decomposition of a polymer. Vapor phase combustion can be controlled by using flame retardants designed for such purposes that decompose at a similar temperature range to that at which the polymer starts to decompose and those radicals are formed, which are less reactive (also known as free radicals). These radicals do not react with the volatile products in the vapor phase and, hence, slow down the combustion process [51]. These free radicals have very low energy and do not have the capability to further prolong the oxidation process. Once the oxidation process is slowed down, the transfer of heat back to the polymer is reduced, which slows down the combustion process, and hence, the flame is eventually extinguished. Typical examples of such cases are the application of organo-bromine and organo-chlorine based flame retardants [52].

### 4.3. Removing Heat of Combustion

**Figure 3:**
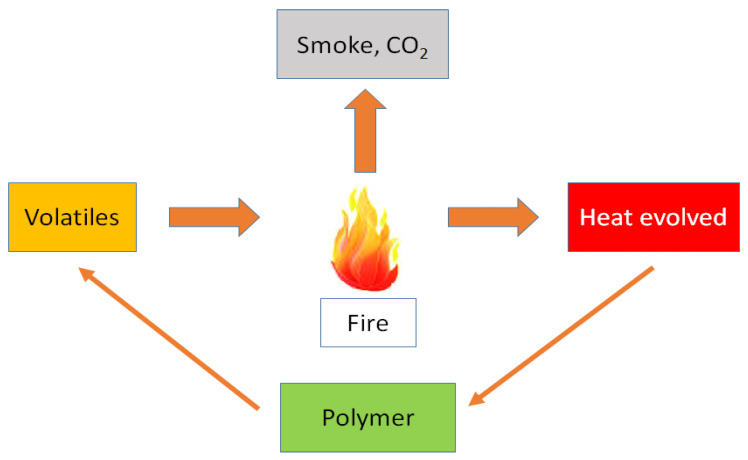
Schematic diagram of a burning cycle.

Due to the burning of volatile compounds in the polymer, heat is generated, and if sufficient heat flows back to the polymer, its thermal decomposition is continued due to the self-sustaining of the burning cycle. The schematic diagram of the burning cycle is shown in Figure 3.

This burning cycle can be broken, and combustion of the polymer can be stopped, if some portion of the heat is eliminated from the burning cycle. By removing heat from the burning cycle, the pyrolysis rate of the polymer is slowed down, and eventually, the combustion process will be stopped [53].

### 4.4. Smoke Suppressants

Due to the burning of some polymeric materials, smoke is generated, which can be very harmful and sometimes can be a cause of death. Polyvinyl chloride (PVC) is one of the polymers that burns with significant amount of smoke, and to reduce its thermal decomposition and smoke emission, smoke suppressants are necessary. Compounds based on zinc, iron, tin, and molybdenum can be used as smoke suppressants in PVC; products such as ammonium octamolybdate and molybdenum trioxide can be used as smoke suppressants in PVC cabling [54].

### 4.5. Char Formation

Volatile products are emitted from the polymer as it is ignited by thermal oxidative reaction, which actually is the main reason for the burning of a polymer. However, this mechanism can be changed to produce less volatile products and more char on the surface of a polymer [55]. The formation of char on polymer surface not only act as smoke suppressant but also removes heat of combustion therefore, this action mechanism is considered far superior than the mechanisms discussed earlier [56]. The type of flame retardants generally preferred for char formation are halogen-free flame retardants, and the system in which they are used is known as intumescent flame retardant system [57].

## 5. Intumescent Flame Retardants

**Figure 4:**
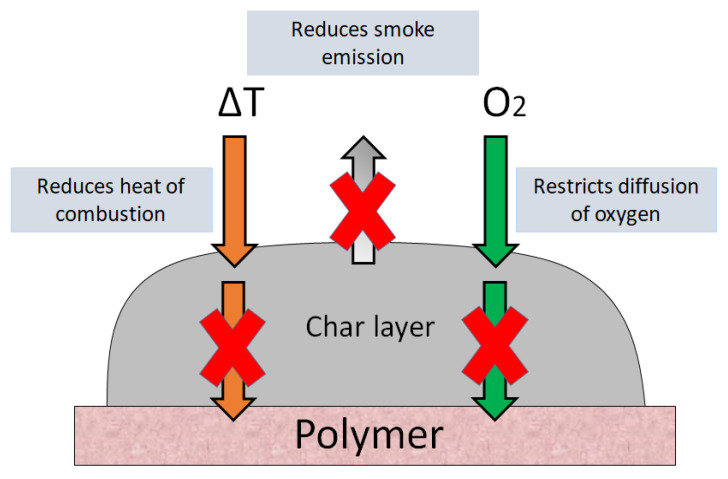
Action mechanism of intumescent flame retardants.

Intumescence originates from the Latin word “intumescere,” which means to swell up [58]. Intumescent material, if heated above a certain temperature, starts to expand and swell up, resulting in the formation of a charred layer on the exterior of the material [59]. This charred layer restricts the diffusion of oxygen to the site of combustion and protects the underlying material from exposure to fire and heat flux [60]. Intumescent flame retardants (IFRs) offer a highly effective strategy to enhance the fire retardancy of polymers as a charred layer develops that acts as a shield between the polymer and heat source and protects the polymer material from further burning and dripping. Modern IFR systems are based on halogen-free flame retardants (HFFRs) which, unlike their halogen-containing counterparts, are environmentally safe, as they do not degrade into dioxins, whereas halogenated compounds with aromatic rings can degrade into dioxins and dioxin-like compounds [61]. Chlorinated dioxins are among the highly toxic compounds listed by the Stockholm Convention on Persistent Organic Pollutants [62]. The action mechanism of IFRs is shown in Figure 4.

The formation of char takes place mostly in the condensed phase, and other than creating a physical barrier, it also has several other benefits, such as not allowing the passage of decomposed volatile products to the place of fire and providing insulation to the underlying material against thermal degradation [63,64]. Since the char forming systems tend to interrupt the burning cycle rather than working on flame poisoning mechanism therefore, regarded as more effective and less hazardous to the environment [65].

Chars normally have broad continuous crust on the outer surface and inner surface have a resemblance to closed foam cell like structure [66]. In an ideal case, the char should be thick and have large volume in order to provide better thermal insulation to the underlying material [67]. In case of intumescent flame retardants better flame retardancy can be achieved at lower loading concentrations (wt %) compared to flame retardants based on magnesium hydroxide and alumina trihydrate [68]. For example, a loading concentration of 15–20 wt % in case of intumescent flame retardants can generate the same level of flame retardancy that can be achieved with 40–50 wt % of magnesium hydroxide and alumina trihydrate [69]. The intumescent system mainly consists of an acid source, which releases acidic species upon heating, and a carbonization agent, which acts as a char former on the material’s surface [70]. Inorganic acids, phosphates, and ammonium salts are used as an acidic source, whereas compounds containing hydroxyl groups such as polyols are used as char formers [71].

## 6. Fire Testing Methods

**Figure 5:**
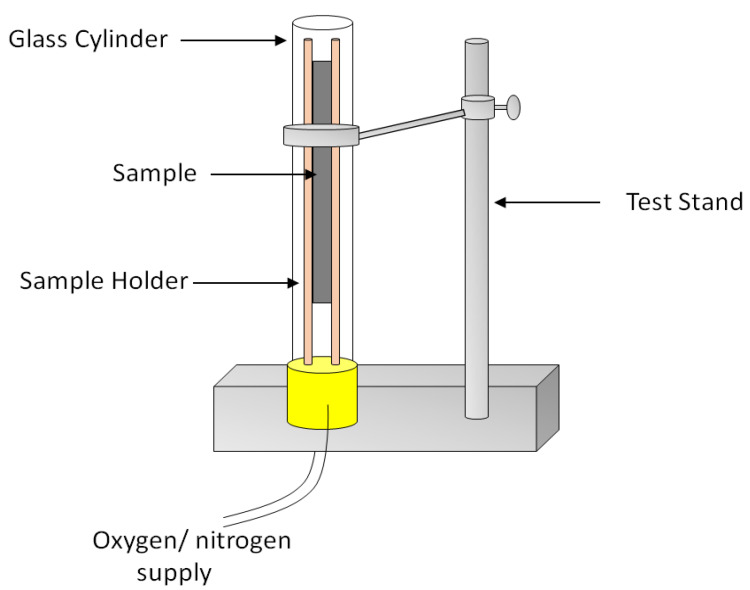
Schematic diagram of limiting oxygen index (LOI) equipment.

The fire hazards of a material can be assessed by various standard testing methods, as each fire regulation defines a particular fire test for its assessment. The fire performance of a material is generally assessed by the following three methods: limiting oxygen index (LOI), UL-94 vertical burning test, and cone calorimetry [72]. LOI measures the minimum concentration of oxygen required to ensure a self-sustained flame. In this test, the specimen is placed vertically in a glass column and is ignited from the top with flame progressing downward. A material with a very low LOI (i.e., around 20%) generally burns quite easily; therefore, higher LOI values corresponds better flame retardancy of a material [73]. This test is performed under the ISO 4589 standard testing method. A schematic diagram for the LOI test is shown in Figure 5.

**Figure 6:**
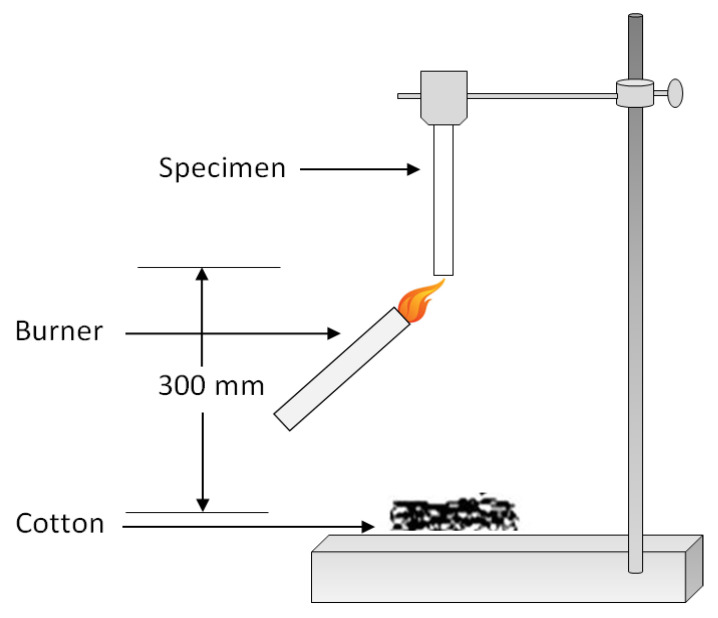
Schematic diagram of UL-94 vertical burning test.

The second commonly used test method in flame retardancy is the UL-94 vertical burning test [74]. In this test, the specimen is positioned vertically and is ignited from the bottom end. The specimens are exposed to flame for 10 s twice, and their flaming time is noted and classified based on three different ratings according to ISO 9773 standard testing method. V-0 is considered to be the best rating, and it corresponds to a specimen that does not drip and flame out in less than 10 s. The V-1 rating corresponds to the specimen, which takes more than 10 s and less than 30 s to flame out and does not drip. The V-2 rating is given to those specimens, which drip after flaming, and take even more time to flame out. No ratings are given to those specimens that do not flame out [60]. The schematic diagram of the UL-94 vertical burning test is shown in Figure 6.

**Figure 7:**
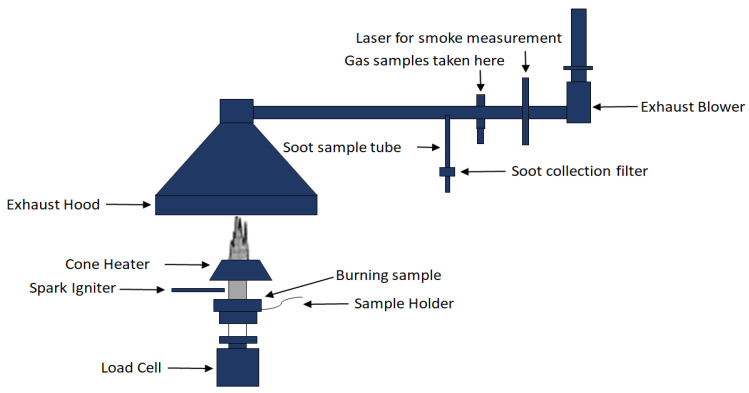
Schematic diagram of cone calorimetry equipment.

A cone calorimeter is a piece of equipment that gives useful insights about the burning behavior of a material by reporting the time to ignition, peak heat release rate, total heat release, and residual mass % of the sample [58]. A specimen in the form of 3–4 mm thick molded sheet is placed horizontally in a controlled heat flux and is ignited with a spark igniter according to the ISO 5660 standard testing method. Heat flux can be varied between 10 kWm^−2^ and 100 kWm^−2^, but it is usually used in the range of 35 kWm^−2^ to 50 kWm^−2^. During this test, the ventilation of air flow is well maintained, and the concentration of oxygen in the air flow is constantly monitored. The intensity of burning is then assessed by measuring the concentration of oxygen in the air flow, as combustion is directly linked to oxygen consumption. Moreover, irrespective of the material, a 1 kg consumption of oxygen is equivalent to a heat release of 13.1 MJ, according to the empirical Huggett relation [75]. The main outcome of this test is the determination of the peak heat release rate of a material, which is a very important parameter to estimate fire hazards. A schematic diagram of the cone calorimetry test is shown in Figure 7.

## 7. Potential Bio-Based and Biodegradable Carbonization Agents

Recently, researchers have shifted their focus toward the development of flame retardants based on biodegradable resources [76]. Carbon is the major element present in biomass, and according to one study, the entire biomass available on Earth represents approximately 560 billion tonnes of carbon [77]. Therefore, carbon element present in natural compounds can be used as a carbonization agent in intumescent flame retardants [78]. In the following section, biodegradable compounds that have the potential to be used as bio-based carbonization agents in intumescent formulations are briefly discussed.

### 7.1. Cellulose

**Figure 8:**
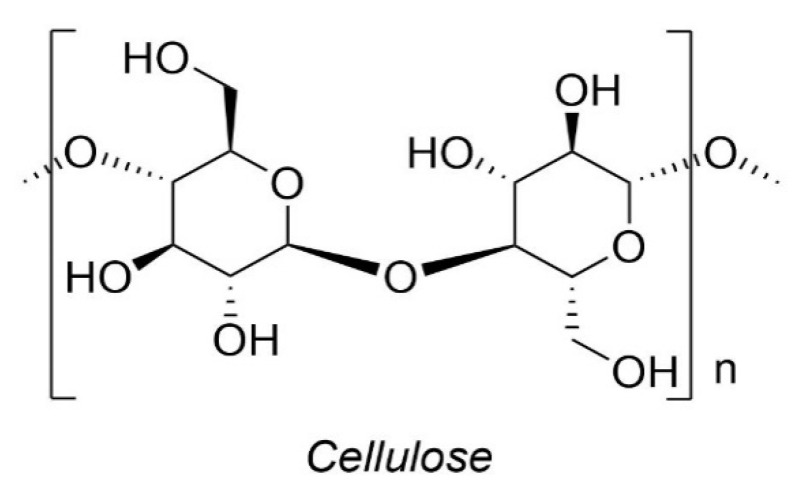
Schematic diagram of cellulose structure [81].

Cellulose is the most abundant natural source of organic compounds on earth and is the core component of the cell walls in plants. It is estimated that, each year on this planet, different plants synthesize approximately 100 billion tonnes of cellulose, which is a linear homopolymer compound, consisting of D-glucose units connected by β 1-4 glycosidic bonds with a polymerization degree ranging between 1000 to 30,000 [79]. During the decomposition of cellulose, aliphatic groups start to disappear, and benzene rings and furan compounds start to increase quite significantly in the condensed phase. At elevated temperatures such as at 800 °C, a char layer is developed. It should be noted that the decomposition route of cellulose can be modified, which is dependent on the presence of other components in cellulose and on the heating conditions [80]. The structure of cellulose is shown in Figure 8.

### 7.2. Starch

**Figure 9:**
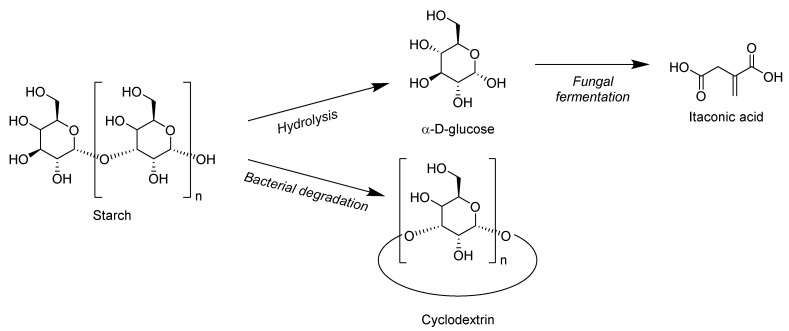
Starch and its derivatives used as flame retardants.

The chemical formula of starch is similar to cellulose, as starch also contains D-glucose units. Plants synthesize this polymer as an energy storage material, and after processing, it takes the form of small granules with a diameter ranging between 1–200 μm. Two different types of macromolecules are present in starch polymer—one is amylose, which is a linear polymer that contains glucose units connected through α 1-4 linkage, whereas the other is amylopectin, which is a branched polymer connected through α 1-6 linkage [82]. The chemical structures of starch and its derivatives are shown in Figure 9.

The production of starch worldwide is approximately 70 million tons per year. By acid or enzyme treatments, native starch can be hydrolyzed into non-complex carbohydrates such as dextrins. Cyclodextrin is one such example, which is obtained by starch degradation by the bacillus amylobacter bacteria [83]. Starch can also be converted into its derivatives by a process called fermentation. For example, glucose or molasses can be converted into itaconic acid by using fungi such as *Aspergillus terreus*. Similarly, tartaric acid can be produced by the fermentation of grape stock to make wine. Both itaconic acid and tartaric acid are used as a raw material for the development of flame retardants [84]. Maqsood et al. [56,85] studied the effect of cornstarch as a bio-based carbonization agent in intumescent formulations as a potential biodegradable substitute for pentaerythritol and PLA/starch composites. The mechanism of intumescence indicating catalytic phosphorylation to produce phosphate esters, which eventually dehydrated the starch and formed char structure containing residue up to 43%, was studied.

### 7.3. Chitosan

**Figure 10:**
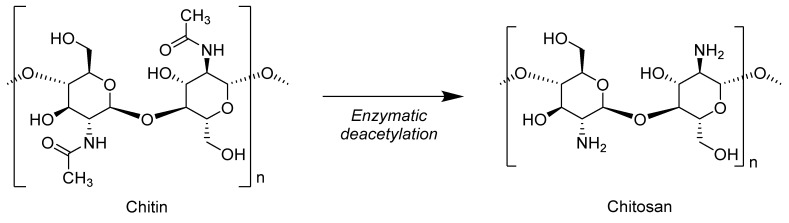
Enzymatic deacetylation of chitin to chitosan.

Chitosan is a copolymer consisting of D-glucosamine and N-acetyl-D-glucosamine units connected through β 1-4 linkage. Chitosan is formed by the enzymatic deacetylation of chitin (Figure 10), which is found in shrimp shells [86].

The acetylation degree of chitosan varies from 60% to 100%, depending upon the commercial application. The production capacity of chitosan worldwide is approximately 20 × 10^3^ tons annually, and its market is growing, especially in North America and Asia. The thermal decomposition of chitosan takes place in three steps. The first weight loss occurs between 130–140 °C, which corresponds to the dehydration of loosely bonded molecules. The second degradation step, which occurs between 260–360 °C, corresponds to the de-polymerization and deacetylation of chitosan. In the third step, due to residual decomposition reaction, a very low weight loss rate occurs above 400 °C, and char residue up to 30 wt % is formed at 500 °C [87].

### 7.4. Alginates

**Figure 11:**
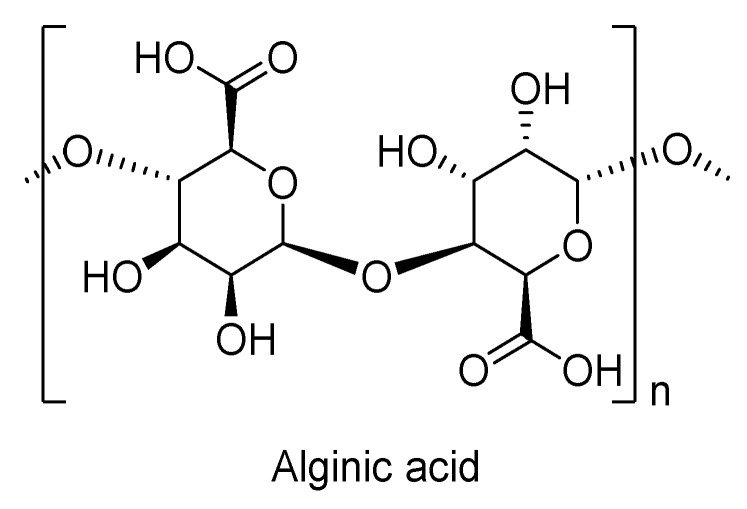
Chemical structure of alginic acid.

Alginates are derived from alginic acid (Figure 11) and are found in the cell walls of brown algae as salts of alginic acid, which is a copolymer of guluronic acid and mannuronic acid with repeating units connected through β 1-4 linkages [88]. The chemical and physical properties of this polymer are dependent on the fraction and distribution of co-monomers present in it. Alginates are considered as anionic polysaccharides. The thermal decomposition of alginates occurs in two main steps under nitrogen atmosphere. In the first step, they undergo dehydration at very low temperatures (100–120 °C) as they contain very high moisture content, i.e., sodium alginate contains about 15 wt % and alginic acid contains about 10 wt %. In the second step, the major decomposition of the polymer starts between 150–300 °C, which leads to char residues of up to 21–25 wt % at a maximum temperature of 800 °C [47].

### 7.5. Lignin

**Figure 12:**
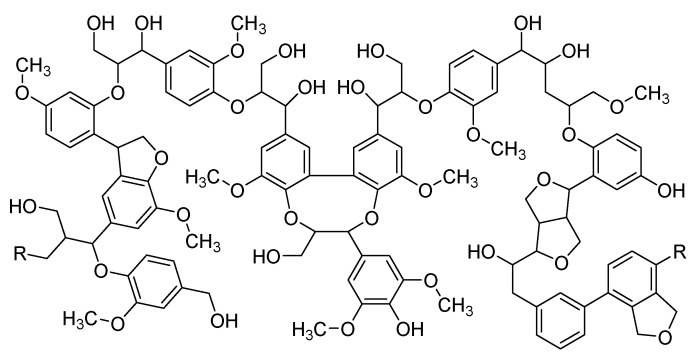
Lignin chemical structure.

Lignin is an aromatic polymer (Figure 12) and is the second most abundant natural polymer (after cellulose). Lignin is present in plants and algae, and its role is quite significant in strengthening the cell walls of plants by providing protection and rigidity. It also gives waterproofness to the cell walls of plants [89]. The thermal decomposition of lignin occurs over a broad range of temperatures (200–500 °C) in two main steps. The decomposition mechanism of lignin is somewhat different compared to other components of biomass. Generally, the first weight loss of lignin occurs between 100–180 °C, which corresponds to the dehydration of water molecules connected to the raw matter [90]. However, the main decomposition starts from 200 °C, and low molecular weight molecules are released by the cleavage from the propanoid side chain. From 275–450 °C, further degradation of lignin takes place due to the cleavage in the main chain either by β-scission, C–C bond, or aryl ether cleavage, which leads to release in large quantity of methane. The condensation of the aromatic structure takes place above 500 °C, which results in the development of a substantial char yield and dihydrogen is released in the gas phase [91]. Maqsood et al. [92] describes the efficiency of kraft lignin (KL) as a bio-based carbonization agent in intumescent formulations, and their FR properties were assessed and compared with conventional carbonization agents. IFR composites comprising different formulations were produced with and without carbonization agents by melt extrusion, and their flammability was assessed by UL-94, LOI, and cone calorimetry tests. Cone calorimetry revealed a 50% reduction in the peak heat release rate of the IFR composites in comparison to 100% PLA and confirmed the development of an intumescent char structure containing residue up to 40% [93].

To conclude the above discussion, the following key-points are the main indicators for the application of bio-based carbonization agents in intumescent flame retardants [94]. The thermal stability of bio-based carbonization agents should be sufficiently high to enable polymer processing, and bio-based carbonization agents should contain functional groups such as hydroxyl and carboxyl groups, which are responsible for char formation by reacting with acid sources in intumescent formulations.

## 8. Approaches Used to Make PLA Flame-Retardant: State of the Art

The flame retardancy of PLA can be improved by various techniques such as blending with more thermally stable polymers and compounding inorganic FRs, nitrogen-based FRs, halogenated FRs, and nanofillers a in PLA matrix. Researchers have tried various techniques to improve flame retardancy of PLA, and some are reported in Table 2.

## 9. Prospects of Bio-Based Flame Retardants for the Industrial Scale-Up

There is a growing interest in the scientific community to develop new bio-based flame retardants due to a variety of biomolecules and green processes being available so that new fire-retardant solutions can be proposed [107]. However, not all the solution strategies proposed at the lab scale can be scaled up to an industrial level. It is hard to forecast the success percentage of different solution strategies due to various factors; however, to monitor the industrial scale up, the three most important factors are health and environmental impact, fire performance, and economic efficiency, which are discussed below.

### 9.1. Health and Environmental Impact

In last couple of decades, the most widely used flame retardants such as brominated FRs have been ruled out due to environmental and health concerns. Most of them were halogenated flame retardants, which have proven to be carcinogenic, neurotoxic and endocrine disruptors. Therefore, for the development of new flame retardants these issues should be addressed, and those approaches should be adopted which do not create health and environmental issues. This is the prime responsibility of European Chemical Agency (ECHA) and REACH regulation to make sure no hazardous chemicals are used in industrial applications and consequently human health and environmental regulations are protected [108].

### 9.2. Fire Performance

Fire performance is another criterion to look for industrial scale up of bio-based flame retardants. The conditions of fire regulations must be fulfilled by new bio-based flame retardants for the applications they are developed for. Generally, three different types of flammability tests are carried out to characterize the fire performance of flame retardants, such as limiting oxygen index (LOI), UL-94 vertical burning test and cone calorimetry. The parameters like, burning rate, time to ignition, heat release rate, char yield, residual mass%, self-extinguishing, smoke emission and effective heat of combustion are extracted from these tests.

### 9.3. Economic Efficiency

The progress in the field of bio-based flame retardants is dependent on the economic efficiency of the developed bio-based compounds. The cost of the raw material and the cost of the processes involved would be crucial as higher cost of the final product could hamper the growth of this sector. The most favorable raw materials could be the one, which are obtained from well- established sectors such as the biomolecules obtained from the wood industry in the form of lignin, cellulose, ligno-sulfonate, vanillin, and carbohydrates that are obtained from renewable resources such as starch and cellulose, as these biomolecules has great potential towards flame retardancy. Lignin can also be obtained as by-product of other plants. The worldwide production capacity of lignin is approximately 50 million tons [109]. Lignin as bio-based carbonization agent is not only effective but also cheaper than other petroleum-based carbonization agents such as Pentaerythritol. Starch is another biomolecule, which is not very expensive. However, chitosan, which is a promising bio-based molecule and is equally effective in flame-retardant applications, has much higher production cost.

## 10. Future Work

A few years ago, no one could have imagined that bio-macromolecules such as carbohydrates (cellulose, starch, chitosan, alginates, etc.), phenolic compounds (lignin, tannins), and others could be used in flame-retardant applications. The related studies have shown that it is possible, at least at the lab and pilot scale, to consider the chemical features of these biomass compounds for the development of biodegradable, non-toxic, environment friendly, and effective flame retardants for different technical applications. Some of these bio-macromolecules can be obtained as a waste material or a byproduct from the wood industry or from agro or food industries, therefore, can present another added value to these industries. Hence, their repossessions and succeeding applications as flame retardants may fulfill the current requirements of valorization of wood, food, and agro industries, thus avoiding their landfill detention. Due to the flame-retardant properties of such bio-macromolecules, their potentiality as an alternative to petroleum-based flame retardants in technical applications is very high, although the approaches discussed in this review still need to be optimized for industrial scale-up. The industrial scale-up of such bio-macromolecules is still under evaluation due to certain limitations—for example, the development of bio-based FR additives at such a large scale is still not practically feasible, and their thermal processing at industrial set-up has not been tested. The other factor that limits their industrial scale-up is the cost of these materials. Undeniably, some of these bio-macromolecules are very expensive—for example, the cost of chitosan (deacetylation degree > 95%) is €190 per kg, although this cost may drop in the next few years due to higher production capacity at the industrial level. In addition, the possibility of exploiting the industrial apparatus for different technical applications used for conventional flame retardants still needs to be tested and validated for such bio-macromolecules as flame retardants. Moreover, in textiles, the washing fastness or the laundering durability of bio-macromolecules-based flame retardants needs significant attention since they have not been very resistant until now [110]. This is due to the kind of additives or coatings that come off after washing and cannot meet specific washing standards even when treated at very low temperature, i.e., 40 °C [111]. Therefore, the durability of bio-based FR–treated fabrics is certainly a big limitation on their ability to be scaled up, since the washing durability of flame retardants is obligatory for most textile applications [112]. Hence, it is necessary to find promising solutions to this limitation without compromising the green features of bio-based flame retardants. One potential solution could be to apply conventional textile finishing treatments to impart washing durability, but that might affect the green features of bio-based flame retardants. To avoid this issue, biologically derived chemical treatments might be one option, but exploiting their potential as a substitute to conventional finishing treatment requires extensive and comprehensive research.

## 11. Conclusions

The bio-based polymers developed from renewable resources, often termed as bioplastics, offer ecological benefits over petrol-based plastics. Due to new governmental legislations hampering the use of hazardous products, their environmental and health issues and fluctuating oil prices have led to the development of products from renewable resources. Therefore, in this review, the potential of bio-based compounds such as cellulose, starch, chitosan, alginates, and lignin as biodegradable carbonization agents in flame retardants for biodegradable polylactic acid (PLA) has been discussed in detail. All these bio-based compounds are known to provide excellent charring effects during combustion, which not only protects the underlying material from further burning but also restricts the diffusion of oxygen and heat transfer. If these bio-based compounds are used together with phosphorous- and nitrogen-based compounds, their efficiency toward flame retardancy can be further improved. However, researchers are working to improve the compatibility of these bio-based compounds with the matrix and to minimize their thermal degradation during melt processing by chemical modifications so that their grafting yields can be increased. There is a need to further optimize the extraction process of these bio-based compounds and their influence on fire retardant behavior before considering them for industrial scale-up because, at this point, their performance at smaller scale is extraordinary; however, still more studies are required to overcome these issues for industrial applications. Moreover, the structure and composition of these bio-based compounds, such as phenolic compounds and carbohydrates, still needs to be studied further in order to overcome major challenges. The thermal stability of these bio-based compounds is another issue that needs to be improved, which can limit their use in some engineering applications.

## Figures and Tables

**Table 1:** Physical and fire related properties of some commercial polymers [28].

Properties	Unit	PLA	PET	PA6	PP
Density	g/cm^3^	1.24	1.38	1.14	0.92
Melting temperature	°C	170–180	255–260	210–220	155–165
Glass transition temperature	°C	55–60	70–80	57–65	−10
Time to ignition	s	58	46	48	42
Smoke generation	m^2^/kg	63	394	195	142
Limiting oxygen index	%	24	19	18	20
Peak heat release rate	kW/m^2^	425–450	625–640	575–590	495–510
Effective heat of combustion	kJ/g	18	24	19	21

PLA = polylactic acid; PET = polyethylene terephthalate; PA = polyamide; PP = polypropylene.

**Table 2:** Approaches used to make polylactic acid (PLA) flame-retardant.

Source	Authors	Short Description	Findings
[95]	Kimura and Horikoshi	Tried to improve flame retardancy of PLA by blending with virgin polycarbonate (PC), but results showed not very significant improvement in flame retardancy of the blend.	To achieve better results, silicon-comprising PC was blended with PLA, but still only a V-2 rating could be achieved in UL-94 vertical burning test.
[96]	Nishida et al.	Studied flame-retardant properties of PLA by compounding Alumina trihydrate (ATH) in the polymer.	However, they found that, in order to get superior results, a relatively higher amount of ATH (about 40 to 50%) needs to be added to the PLA matrix.
[97]	Yanagisawa et al.	Incorporated ATH together with phenolic resins in PLA matrix to improve its flame retardancy for electronic applications. A significant char formation during combustion on the surface of the composite was observed.	The addition of phenolic resin not only resulted in improved flame retardancy, reinforced by alumina from ATH on the surface of composite, but also reduced the loading content of ATH to 35% (*w*/*w*).
[98]	Kubokawa et al.	Determined flame retardancy of PLA based fabrics by using bromine containing additives, and tri-phenyl phosphates and tested LOI of the fabrics.	Limiting oxygen index (LOI) of untreated fabrics was 24%; however, after treatment with FR additives, LOI of the fabrics increased to 28% with tri-phenyl phosphate and to 26% with bromine-containing FR additives.
[99]	Wang et al.	Prepared composites containing pentaerythritol (PER) and melamine cyanurate (MC) by controlling the weight ratio of 2:2:1 and organo-modified zinc-aluminum-layered double hydroxide (Zn-Al-LDH).	Microscale combustion calorimeter (MCC) and cone calorimetry results revealed substantial progresses in flame retardancy of the nanocomposites. A significant reduction in heat release rate and total heat release was observed.
[100]	Wei et al.	Investigated the effect of aryl poly-phenyl phosphonate together with PLA. LOI, UL-94 vertical burning and cone calorimetry tests were carried out together with the investigation of thermal and mechanical behavior of the composites.	PLA composites containing 7 wt % and 10 wt % of poly phenyl phosphonate achieved V-0 rating in UL-94 vertical burning test. However, not much improvement in HRR and THR of the FR composites were observed in comparison to neat PLA.
[101]	Bourbigot et al.	Studied flame-retardancy of different polymer nanocomposites such as polylactides, polyurethane and polyamides. Different nano-fillers such as carbon nanotubes and organoclay were incorporated in these polymers and their flame retardancy was investigated.	Found that nano-dispersion play a vital role in improving flame retardancy of nanocomposites and nano-fillers give better results when they are used in combination with inorganic flame retardants due to better synergistic effects.
[102]	Solarski et al.	Developed PLA/clay nanocomposites and studied their thermal and fire properties. Four different formulations ranging from 1 wt % to 4 wt % of the organo clay (C30B) were prepared. The nanocomposites were melt spun to produce multifilament yarns.	Yarns with better mechanical properties were used to produce knitted structures. The fire properties of the knitted fabrics were tested by cone calorimetry. It was observed that pHHR of the fabrics containing only 2 wt % of the clay were reduced up to 38%.
[17]	Suardana et al.	Prepared bio-composites containing natural fibers together with di-ammonium phosphate and investigated their mechanical and fire related properties.	By increasing wt % of di-ammonium phosphate, fire properties, flexural modulus, and weight loss rate of the composites were improved; however, tensile and flexural strengths of the composites were reduced.
[59]	Qian et al.	Prepared aluminated mesoporous silica and used it as an FR additive in PLA resin. Different formulations of fumed silica and aluminated mesoporous silica were compounded in PLA resin.	Achieved UL-94 V-0 rating and LOI value also increased quite significantly. pHHR of FR composites decreased to about 15% compared to neat PLA by the addition of only 0.5 wt % of aluminated mesoporous silica.
[103]	Wang et al.	Developed an inherently fire-resistant PLA polymer using a chain-extending procedure of pre-polylactic acid (PPLA). PPLA was produced by direct condensation reaction of L-lactic acid and its fire properties were tested.	Here, 5 wt % of PPLA in PLA polymer would be sufficient to achieve remarkable FR properties as LOI value of 35% and UL-94 V-0 rating was achieved with delayed ignition time compared to pure PLA.
[104]	Mngomezulu et al.	Investigated FR properties of PLA and expandable graphite (PLA/EG) composites. EG was compounded in PLA with different wt % to produce PLA/EG composites and their surface morphology, filler dispersion, dynamic mechanical behavior and crystallization rate were studied.	The presence of graphite layers with not so good filler dispersion resulted in poor bonding between PLA resin and EG. The crystallization rate of PLA was increased with an increase in the glass transition temperature. Furthermore, lower modulus of the composites with higher wt % of EG was observed.
[105]	Shumao et al.	Used ramie fibers together with PLA to reinforce the polymer and to enhance the mechanical properties. Used different formulations together with ramie fibers.	At 40 wt %, loading the LOI value could only be reached at 30%. Resulted phosphoric acid contributed in intermolecular dehydration of ramie fibers followed by dehydrogenation.
[63]	Zhan et al.	Investigated the combustion and thermal degradation behavior of PLA with a special flame-retardant consisting of spirocyclic-pentaerythritol bisphosphorate disphosphoryl melamine (SPDPM) and melt compounded with different wt % in PLA.	Attained UL-94 V-0 rating at 25 wt % loading and LOI value was 38%. A significant reduction in weight loss rate of PLA was observed after addition of SPDPM in PLA as confirmed by thermogravimetric analysis.
[106]	Fox et al.	Polyoligomeric Silsesquioxane (POSS) modified cellulose was melt blended with PLA to prepare PLA/POSS composites. Thermal and fire related properties of as prepared composites were tested by thermogravimetric analysis, dynamic mechanical analysis and cone calorimetry.	pHHR of the composites was reduced to 45% by the introduction of 15 wt % of modified cellulose in comparison to pHHR of neat PLA. Total heat release (THR) was also reduced to 20% and lesser smoke generation was observed in comparison to non-modified cellulose.
